# The Protective Role of Probiotics against Colorectal Cancer

**DOI:** 10.1155/2020/8884583

**Published:** 2020-12-09

**Authors:** Sujuan Ding, Chao Hu, Jun Fang, Gang Liu

**Affiliations:** College of Bioscience and Biotechnology, Hunan Agricultural University, Hunan Provincial Engineering Research Center of Applied Microbial Resources Development for Livestock and Poultry, Changsha, Hunan 410128, China

## Abstract

Colorectal cancer (CRC) is the fourth leading cause of cancer-related deaths worldwide and a major global public health problem. With the rapid development of the economy, the incidence of CRC has increased linearly. Accumulating evidence indicates that changes in the gut microenvironment, such as undesirable changes in the microbiota composition, provide favorable conditions for intestinal inflammation and shaping the tumor growth environment, whereas administration of certain probiotics can reverse this situation to a certain extent. This review summarizes the roles of probiotics in the regulation of CRC, such as enhancing the immune barrier, regulating the intestinal immune state, inhibiting pathogenic enzyme activity, regulating CRC cell proliferation and apoptosis, regulating redox homeostasis, and reprograming intestinal microbial composition. Abundant studies have provided a theoretical foundation for the roles of probiotics in CRC prevention and treatment, but their mechanisms of action remain to be investigated, and further clinical trials are warranted for the application of probiotics in the target population.

## 1. Introduction

The global incidence of CRC is very high and continues to increase every year. Data show that CRC accounts for approximately 9% of all cancer-related deaths and is the third leading cause of death in women after breast cancer and the second leading cause of death in men after lung and prostate cancers [1, 2]. Despite advances in screening and early diagnosis of CRC, CRC remains the second leading cause of cancer-related deaths. Therefore, more research attention to CRC prevention, treatment, and prognosis is crucial.

Recent evidence has demonstrated that probiotics may contribute to the treatment of CRC [3]. According to the definition established in 2002 by the Food and Agriculture Organization of the United Nations (FAO) and the World Health Organization (WHO), probiotics are “live microorganisms which when administered in adequate amounts confer a health benefit on the host” [4]. Several studies have highlighted the critical role of probiotics in regulating intestinal disorders, such as diarrhea [5], inflammatory bowel disease [6], irritable bowel syndrome [7], *Helicobacter pylori* infection [8], and lactose intolerance [9]. Probiotics can also inhibit the development of CRC by modifying the intestinal microbial composition, intestinal epithelial system, and intestinal immune responses. *Akkermansia muciniphila* (AKK), an intestinal symbiotic bacterium living in the mucosal layer, has been shown to exhibit a high antitumor efficacy with favorable clinical outcomes [10, 11]. One study demonstrated that AKK initiates an antitumor immune response by activating the Toll-like receptor signaling pathway through its outer membrane protein Amuc. Meanwhile, it is found that the administration of AKK together with interleukin- (IL-) 2 protects the intestinal barrier function, suggesting a new therapeutic strategy for CRC [12].

## 2. Interaction between Probiotics and the Host

Probiotics used in foods are safe for human consumption, with most being certified as Generally Regarded as Safe (GRAS) by the U.S. FDA or as Qualified Presumption of Safety (QPS) by the E.U. EFSA [13]. Recent studies based on animal models and clinical interventions have demonstrated the critical role of probiotics in the prevention and treatment of several human diseases [14]. The interplay between probiotics and the human gastrointestinal tract (GIT), comprising the mucus layer, epithelial layer, and gut-associated lymphoid tissue, influences the disease process in the human host [15]. The mucosal layer of the intestinal tract comprises a loose outer sublayer of gel-forming mucins and a dense inner sublayer of mucins. The outer sublayer is relatively abundant with bacteria, antimicrobial peptides, and immunoglobulin, whereas the inner sublayer has few or no microbes [16, 17]. The secondary interaction between probiotics and the intestinal tract occurs in the intestinal epithelial layer containing different cell subgroups and spanning across the entire intestinal cavity. The main functions of this layer are absorption of nutrients, secretion of mucin, and release of antimicrobial molecules such as defensin and lysozyme [18]. Bacteria affect the intestinal epithelial barrier function through pattern recognition receptors [19]. Probiotics interact with host intestinal epithelial cells (IECs) by adhering to the intestinal wall and stimulating the production of mucus, thereby enhancing the intestinal barrier [20]. Through such interaction, probiotics compete with pathogenic bacteria for niche occupancy [21], prevent pathogenic bacteria from growing and proliferating in the intestine by competing with them for nutrition and energy [22, 23], and reduce intestinal pH by fermenting dietary fiber to produce short-chain fatty acids (SCFAs) [24].

## 3. Colorectal Cancer

CRC causes nearly 700,000 deaths every year, making it the most fatal cancer in the world after lung cancer, liver cancer, and gastric cancer [25]. Unhealthy eating habits, especially frequent consumption of low-fiber and high-fat foods characteristic of the Western diet, are crucial factors in the development of intestinal disorders [26], which suggests that the prevalence of the Western diet and lifestyle also increases the incidence of CRC. CRC is a slow-developing disease, and survival rates have improved in recent decades owing to the improvements in preventive cancer screening, which allows early detection. Screening thus remains the mainstay for CRC prevention [27]. CRC is believed to be associated with aging, and the majority of people who undergo regular screening for CRC are older than 50 years; this underestimates the likelihood of CRC in younger patients, even when they present with abdominal pain and bloody stools [28].

Further advancements in the prevention and treatment of CRC warrant a complete understanding of the normal biology of the colon and the pathogenesis of CRC. The basic unit of the colon includes crypts and luminal surfaces. When the intestine is in a state of homeostasis, each colon crypt contains 14–16 pluripotent stem cells marked with the transmembrane protein leucine-rich repeat-containing G protein-coupled receptor 5 (LGR5). These stem cells can produce all differentiated cell types in the colon cavity [29, 30]. LGR5^+^ stem cells can produce rapidly proliferating transit-amplifying (TA) cells, which account for approximately two-thirds of the crypts. TA cells mainly differentiate into four cell types, namely, absorbable IECs, goblet cells, cluster cells, and intestinal endocrine cells, which are renewed approximately once a week [31]. The main transcription target of the Wnt pathway in intestinal crypt stem cells is the serpentine transmembrane receptor LGR5, which inhibits the expression of the oncogene *Myc* and of the basic helix-loop-helix (bHLH) transcription factor achaete-scute like 2 (ASCL2), which is associated with stem cell self-renewal [32]. Mutations in the adenomatous polyposis coli (*APC*) gene are the potential cause of familial adenomatous polyposis, known as hereditary colon cancer syndrome [33, 34]. APC loss is also the major driver of Wnt signaling in CRC [35]. Evidence indicates that different *APC* mutations result in different levels of Wnt signaling pathway activity, which is related to the typical tumor location in the large intestine [36, 37].

## 4. Gut Microbiota

The human gut microbiota is a rich, diverse, and complex microbial community composed of fungi, bacteria, archaea, viruses, bacteriophages, and protozoa living in a symbiotic relationship with the human host [38]. The composition and activity of the gut microbiota is a hot topic in the cross-research field of human microbiology and health, and it is directly related to the study of probiotics [15]. The commensal bacteria form a tight and complex interaction network with their hosts and are involved in protecting the gut from harmful substances [39]. Metagenomic evidence suggests that the gene set of different gut microbial species pools and the functional prediction of the community are the same and similar, respectively, among individuals. However, the composition and function of the gut microbiota vary with diet, location, sex, age, and race [40, 41]. Diet is the main regulator of the intestinal microbial function. In general, the ratio of the phyla Firmicutes/Bacteroidetes is higher in individuals following a Western-style diet, whereas the abundance of the genus *Prevotella*, belonging to the Bacteroidetes phylum, is higher in individuals following a subsistence diet [42–45]. In healthy individuals, more than 90% of the ingested diet is absorbed by the small intestine, whereas the complex carbohydrates that pass undigested from the small intestine, such as fiber, protein residues, and primary bile acids secreted by the body in response to fat intake, are digested in the colon [46]. These components of the diet influence the composition and function of the gut microbiota. Saccharolytic fermentation of complex carbohydrates by the colonic bacteria produces SCFAs, with acetic, propionic, and butyric acids (in a molar ratio of 3 : 1 : 1) accounting for approximately 90%–95% of colonic SCFAs [47, 48]. Butyrate regulates mucosal inflammation and antitumor activity by participating in intestinal microbial balance, proliferation inhibition, immune regulation, and epigenetic regulation [49].

The gut microbiota is composed of more than 1,000 bacterial species, including beneficial and pathogenic microbes, and is dominated by Firmicutes and Bacteroidetes. In healthy individuals, the beneficial microbes surpass the pathogenic microbes and inhibit their excessive growth [50]. The gut microbiota can thus be considered as an “organ” that performs significant roles, including the utilization of complex dietary constituents, anabolism of various important compounds, regulation of immune function, and maintenance of intestinal barrier integrity [51]. Hence, the role of the gut microbiota in the pathogenesis of intestinal disorders cannot be underestimated, and its role in the pathogenesis of CRC has received much attention in recent years [52]. Whether microbiota dysbiosis is the cause or result of CRC is still unknown, which remains a foundational issue in understanding CRC [25]. The occurrence of CRC is usually closely related to the mucosal microbes near the site of tumorigenesis [53–55]. The main bacterial species that influence the development of CRC are not yet completely clear, but the available evidence suggests that the abundances of *Fusobacterium nucleatum* (Fn), *Escherichia coli*, *Helicobacter pylori*, and *Bacteroides fragilis* are closely associated with CRC [56]. It is also suggested that a decrease in bacterial diversity is related to the occurrence of tumors, but its role in tumorigenesis remains to be confirmed in further studies [57].

## 5. Mechanism Underlying the Role of Probiotics in the Regulation of CRC

**Figure 1:**
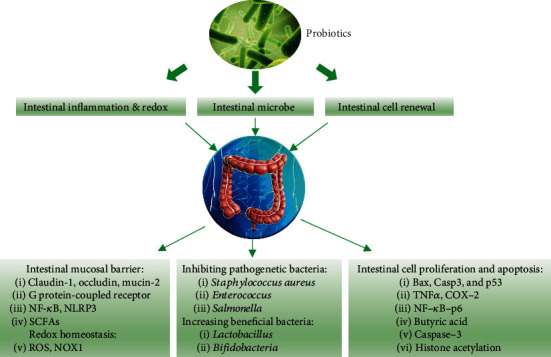
The various roles of probiotics in colorectal cancer prevention and treatment.

Research on bioactive components and gut microbes has revealed that probiotics may play an important role in cancer prevention and treatment in addition to regulating the homeostasis and immune state of the intestinal epithelial system [58]. Multiple mechanisms have been hypothesized for the CRC-preventive and therapeutic effects of probiotics. For example, at the level of intestinal ecology, probiotics may reduce the number of pathogenic bacteria in the gut by competing with the pathogenic bacteria for intestinal niche occupancy or reduce the level of carcinogens [59]. In addition, SCFAs produced by microbial metabolism could stimulate the proliferation and differentiation of intestinal cells in the large and small intestines [60]. For instance, intestinal acetic acid produced by *Propionibacterium* can trigger the release of cathepsin D into the cytosol of cancer cells by increasing the permeability of their lysosomal membrane, thereby protecting the cells from apoptosis [61]. In this section, we focus on the various roles of probiotics, including enhancing the intestinal mucosal barrier, reducing intestinal inflammation, inhibiting the activity of pathogenic bacteria, regulating redox homeostasis, and reprogramming the composition of microorganisms, in the regulation of CRC (Figure 1).

### 5.1. Enhancing the Intestinal Mucosal Barrier

The complete intestinal mucosal barrier includes physical, chemical, biological, and immune barriers. In a healthy state, the intestinal barrier can protect the gut from toxins and pathogens [62]. Probiotics stimulate mucus secretion by IECs, which functions as a barrier between the mucosa and microorganisms that prevents the translocation of bacteria and toxins and also inhibits the adhesion and invasion of pathogenic bacteria in IECs [63]. Probiotics enhance the intestinal barrier by regulating the expression of tight junction proteins, such as claudin-1 and occludin, and stimulating intestinal cells to suppress inflammation and accelerate epithelial cell remodeling by promoting mucin secretion [64–66]. Occludin is a transmembrane tight junction protein that forms the mechanical barrier of epithelial cells, and the level of occludin is a functional indicator of the intestinal mechanical barrier [67]. *Bifidobacterium infantis* and *Lactobacillus acidophilus* were found to protect intestinal permeability by regulating the expression of occludin and claudin-1 proteins and protecting the activation of nuclear factor kappa-B (NF-*κ*B) induced by IL-1*β* in Caco-2 cells [68]. *Lactobacillus plantarum* ZLP001 reversed the decrease in claudin-1 and occludin protein levels induced by enterotoxigenic *E. coli* and decreased the levels of the inflammatory cytokines IL-6, IL-8, and tumor necrosis factor alpha (TNF-*α*) [69]. Mucin-2 glycoprotein (MUC2) formed by goblet cells in the form of a disulfide cross-linked network is the main component of colonic mucus [70]. Muc2 gene inactivation in mice has been shown to increase close contact between bacteria and IECs, leading to inflammation and eventually colon cancer [71]. SCFAs produced by microbes through fermentation of complex carbohydrates can enhance barrier function by G protein-coupled receptor-mediated sensitization of the IEC inflammasome and reducing the oxygen concentration of IECs to induce hypoxia-inducible factors [56].

### 5.2. Reducing Intestinal Inflammation

Immunotherapy involves the stimulation of innate immunity and the subsequent activation of antitumor immune responses [72]. Evidence suggests that the mechanism of inflammation is a driver of tumor maturation and that inflammation is closely associated with the risk of CRC [73]. The gut microbiota plays an important role in the formation of an inflammatory microenvironment, and the occurrence of inflammation in turn affects the composition of the gut microbiota. Intestinal tumorigenesis is driven by inflammation, microbes, and immunity [74]. Probiotics contribute to the normal functioning of the immune system and affect the host immune status by participating in the differentiation of immune cells and stimulating the production of anti-inflammatory substances, antioxidants, and antitumor components [66, 75, 76]. The colonic immune system contains many types of immune cells, with macrophage being one of the most abundant immune cell types [77–79]. A possible mechanism by which probiotics improve the stability of the colonic environment is by acting on the colonic macrophages [80]. Macrophages perform probiotic phagocytosis in a strain-dependent manner and prevent deep tissue destruction after infection by secreting anti-inflammatory mediators [60]. Evidence has revealed that the interaction between probiotics and Toll-like receptors expressed on IECs leads to the production of TNF in the cells, which inhibits NF-*κ*B in macrophages and stimulates the production of IL-8 required for neutrophil production [81]. A study showed that heat-killed *Enterococcus faecalis* could reduce caspase-1 activity and IL-1*β* maturity, thereby achieving consistent activation of the NLRP3 inflammasome in macrophages [82]. Furthermore, SCFAs produced by dietary fiber fermentation are not only the main energy source for IECs but also the regulator of the intestinal immune response [83]. Mechanistically, the induction of a tumor phenotype may be due to the proliferation of colon epithelial cells induced by butyrate. However, butyric acid and its receptor GPR109A can also inhibit colitis and tumorigenesis, indicating that butyrate has anticancer potential [84].

### 5.3. Regulating the Generation of Reactive Oxygen Species

Oxidative stress plays a vital role in the occurrence of CRC [85]. Reactive oxygen species (ROS) are by-products of normal cell metabolism in the GIT. The control of redox homeostasis by the intestinal epithelium, that is, the balance between antioxidation and oxidative stress, is a vital factor affecting intestinal functions such as digestion and absorption of nutrients, immune response, stem cell proliferation, and apoptosis of apical enterocyte [86–88]. ROS and its oxidation products may damage the antioxidant system of intestinal tissues and destroy the normal function of the intestine, potentially leading to intestinal mucosal hyperplasia [89–91]. DNA mutations caused by ROS are thought to be involved in the early inflammatory process of CRC development [92, 93]. Nicotinamide adenine dinucleotide phosphate oxidase (NOX), expressed on the surface of inflammatory phagocytes such as neutrophils and phagocytes, participates in ROS generation. It is also involved in the proliferation and invasion of epithelial tumor cells. ROS produced by NOX1 can in turn trigger angiogenesis in the epithelial tumor cells by inducing angiogenic factors, thus promoting their vascularization and proliferation [94, 95]. Gut microbial dysbiosis caused by the mucosa-associated immune system may promote leukocyte-induced inflammation and oxidative overreaction, consequently aggravating intestinal mucosal injury [96]. Of the colonic commensal bacteria considered to play a crucial role in CRC development, enterotoxigenic *Bacteroides fragilis* (ETBF) is suggested to cause inflammatory diarrhea by secreting toxins [97]. *B. fragilis* toxin promotes the production of ROS in IECs and dendritic cells [98, 99]. A study showed that commensal bacterial rapidly produced ROS on IECs both in vitro and in vivo and caused oxidative inactivation of the catalytic cysteine residue of Ubc12, resulting in the suppression of the cullin-1 ubiquitination and the consequent inhibition of NF-*κ*B and *β*-catenin signaling pathways [100].

Research on the role of the gut microbiota in regulating gastrointestinal redox homeostasis is still in its infancy. However, some preliminary data have uncovered the relationship between the microbiota and redox status, which plays an important role in the regulation of gastrointestinal health. Evidence suggests that the hosts' ROS is associated with the balance of the gut microbial composition; for instance, the oxidation state of the host is negatively correlated with the abundance of *Lactobacillus* and *Bifidobacterium* and positively correlated with that of *E. coli* [101]. Findings from mouse models have indicated that a high abundance of Bacteroidetes in the colon controls pathogen loads by inducing proinflammatory and prooxidative reactions, which play a key role in preventing intestinal infections [102]. The results of a study in a mouse model of CRC induced by azoxymethane showed that the structure of the intestinal microbiota was regulated by *Clostridium butyricum* administered by gavage, which involved a reduction of the ratio of Firmicutes/Bacteroidetes, an increase in the relative abundance of probiotics, an increase in tumor cell apoptosis, inhibition of the NF-*κ*B pathway and IL-6 levels, and a reduction in CRC incidence [103]. In one study, the supernatants of *Musa paradisiaca* inflorescence fermented with *Lactobacillus casei* and *Bifidobacterium bifidum* were found to induce DNA damage, promote ROS generation, and initiate the apoptosis signaling pathway in HT-29 colon cancer cells [104]. Another study showed that *Lactobacillus paracasei* subsp. *paracasei* M5L suppressed HT-29 cell proliferation and could promote HT-29 cell apoptosis through ROS production and calreticulin translocation [105]. Moreover, *Lactobacillus* can exert anticancer effects by producing antioxidants such as glutathione, superoxide dismutase, and catalase, suppressing inflammation and tumor size, and inhibiting the expression of tumor-specific proteins and polyamine components. However, the mechanism of the anticancer effect of *Lactobacillus* in relation to CRC needs to be investigated further [106–108].

### 5.4. Inhibiting the Enzyme Activity of Pathogenic Bacteria

Endogenous toxic compounds, such as N-nitroso, cresol, aglycones, and phenols, promote the development of CRC by participating in antiapoptotic pathways in the intestine. The carcinogenic effects of endogenous toxic and genotoxic compounds in the intestinal microenvironment may be further influenced by pathogenic bacterial enzymes such as 7-*β*-dehydroxylase, nitroreductase, *β*-glucuronidase, *β*-glucosidase, and azoreductase [109, 110]. For example, pathogenic bacteria such as *Staphylococcus aureus*, *Enterococcus*, and *Salmonella* synthesize azoreductase, which metabolizes dyes and drugs to generate toxic aromatic amines [111]. Polyketide synthase (pks) islands present in some strains of *E. coli* encode the genotoxin colicin, which can induce single-stranded DNA breaks [112]. Furthermore, the DNA damage response signaling pathway activated in infected cells tends to increase the mutation rate [113]. Enterotoxigenic *B. fragilis* has been reported to participate in CRC initiation by producing a toxin [114]. Nevertheless, studies have shown that probiotic supplementation may suppress the activity of bacterial enzymes [115, 116]. For example, *Lactobacillus* could suppress the dehydrogenation of *L. rhamnosus* GG (LGG) and reduce the level of primary bile acid by reducing the activity of *β*-glucuronidase [117]. Animal model studies have shown that yogurt starter bacteria could reduce the activity of bacterial enzymes, which may be the mechanism underlying the CRC-preventive effects of probiotics [118]. However, in healthy subjects, *L. acidophilus* A1, *L. plantarum* 299V, and *L. rhamnosus* DR20 could not decrease glucuronidase activity [119, 120].

### 5.5. Regulating the Proliferation and Apoptotic Responses of CRC Cells

Apoptosis plays a key role in regulating the number of cells by balancing cell renewal and eliminating mutant cells, which is one of the main mechanisms of tumor cell death in CRC. The decrease in apoptosis is an important disease event and is accompanied by disruption of cell proliferation regulation [121]. Therefore, apoptotic pathways are a promising target for disease prevention and treatment to manage cell survival and death through apoptosis regulation. Accumulating evidence has highlighted the critical role of probiotics in the regulation of cell proliferation and apoptosis, which may thus be a vital therapeutic and preventive measure against CRC [122]. In rat models, LGG decreased the incidence and size of dimethylhydrazine-induced tumors while inhibiting the expression of inflammatory proteins, namely, TNF-*α*, COX-2, and NF-*κ*B–p6, reducing the expression of the antiapoptotic protein Bcl-2, and increasing the expression of the proapoptotic proteins Bax, Casp3, and p53, suggesting that LGG has the potential to prevent colon cancer [123]. In another study, *L. plantarum* DY-1 showed a strong antiproliferative activity in an HT-29 cell model that involved retarding the development of the cell cycle from G0-G1 phase to G2-M phase and induction of cell apoptosis possibly via caspase-3, indicating that *L. plantarum* DY-1 has antitumor potential [124]. In addition, SCFAs reduce cancer risk by reducing tumor growth and activating apoptosis cascades via hyperacetylation of histones [125]. *Propionibacterium freudenreichii*, a probiotic in the human gut microbiota, has been found to suppress colorectal adenocarcinoma cells via SCFA-mediated apoptosis [126]. Butyric acid was found to prevent CRC by regulating the cell cycle, differentiation, and apoptosis of colon cancer cell lines [127–129].

### 5.6. Reprogramming the Composition of Gut Microbes

The ultimate goal of probiotic intervention is to exert regulatory effects, including immune regulation, immune barrier strengthening, and regulation of the gut microbial composition, against certain disorders [15]. Changes in the gut microbial composition are inextricably linked to the development of CRC. Substantial evidence from animal model studies suggests that probiotics, such as *Lactobacillus* and *Bifidobacterium*, have significant effects on intestinal microbial composition [130, 131]. The colon is teeming with microbes, and this large population is mostly benign, but some are pathogenic bacteria, and the increase in the abundance of these pathogens in the colon is associated with acute or chronic conditions, such as obesity, inflammatory bowel disease, and CRC [132]. *E. coli* is an intestinal symbiotic bacterium, and certain strains of it can promote intestinal inflammation leading to the production of colicin, a potential carcinogen [133]. Pathogenic *E. coli* exists in CRC tissues and is thus used as a marker in tumor staging and prognosis [134]. Furthermore, as noted earlier, *E. coli* containing pks islands, which encode colibactin, can induce single-stranded DNA breaks, and thus, changes in the *E. coli* gene set influence the phenotype of the disease [112, 135]. Compared with mice injected with *E. coli*, those injected with *Bacillus polyfermenticus* showed reduced tumor size, while HT-29 cells injected with *B. polyfermenticus* showed reduced expression of ErbB2 and ErbB3 at the protein and mRNA levels [136, 137]. Intestinal pathogenic microbes such as *Bacteroides* and *Clostridium* are associated with the pathogenesis of CRC [138]. A double-blind test of synbiotics (LGG, *Bifidobacterium lactis* Bb12, and oligofructose) in 37 patients with CRC and 43 colonic polypectomy patients demonstrated that the abundance of *Lactobacillus* and *Bifidobacterium* increased, whereas that of *Clostridium perfringens* decreased in CRC patients, and synbiotic intervention inhibited the colorectal cell proliferation ability and colon cell necrosis ability and improved epithelial cell barrier function in colonic polypectomy patients [139].

## 6. Perspectives

Although certain bacterial species are classified as probiotics due to their benefits to the host health, changes in host health status require the regulation of specific probiotic bacteria rather than the probiotic community in the gut. Substantial research has explored the role of probiotics in the prevention, treatment, and prognosis of CRC. Such dedicated research has revealed a variety of regulatory roles of probiotics, such as enhancing the immune barrier, regulating the intestinal immune state, inhibiting pathogenic enzyme activity, regulating CRC cell proliferation and apoptosis, and regulating the intestinal microbial composition. Although the evidence from clinical or animal model experiments has provided a theoretical foundation for the application of probiotics, evidence from clinical trials on the benefits of probiotics in the prevention and treatment of CRC is lacking. Therefore, further clinical trials are warranted to explore the mechanisms of probiotics in the regulation of CRC. In addition, it remains unknown whether gut microbial dysbiosis is the cause or result of CRC. To address this knowledge gap, further studies on the interactions between probiotics and intestinal microorganisms in CRC development are warranted. Meanwhile, although the gut microbiota contains fungi and viruses in addition to bacteria, there is little evidence supporting the role of fungi and viruses in the gut microbial dysbiosis leading to CRC development.
